# Anatomy and Complications Related to Ligation of the Left Gastric Vein During Pancreatectomy: A Systematic Literature Review

**DOI:** 10.7759/cureus.89070

**Published:** 2025-07-30

**Authors:** Lydia Renardson, Ali Yasen Mohamedahmed, Rami Ayoub, Vandana B Giriradder, Hassaan Bari, Jawad Ahmad

**Affiliations:** 1 Department of Hepatobiliary Surgery, University Hospital Coventry and Warwickshire, Coventry, GBR; 2 Department of General Surgery, University Hospitals of Derby and Burton NHS Trust, Burton on Trent, GBR

**Keywords:** anomalies of vascular anatomy, a systematic review, : delayed gastric emptying, distal pancreatectomy(dp), hepatic-bilio-pancreatic surgery, left gastric, post-op complications, preoperative screening, total pancreatectomy

## Abstract

Pancreatectomy remains the gold standard treatment for pancreatic malignancies but is frequently complicated by delayed gastric emptying (DGE) and gastric venous congestion (GVC). Disruption of the left gastric vein (LGV) has been increasingly implicated in these postoperative complications. We conducted a systematic review, in accordance with Preferred Reporting Items for Systematic Reviews and Meta-Analyses (PRISMA) 2020 guidelines, to assess the impact of LGV preservation on post-operative complications. A comprehensive search of PubMed, MEDLINE and Google Scholar identified 205 records, 12 of which met the inclusion criteria. This comprised five case reports, two case series, two retrospective studies, one prospective imaging study, one single-centre prospective observational study and one retrospective case series. Six studies described intraoperative LGV identification via imaging or direct visualisation, while six reported complications following LGV sacrifice. Selected cases involved LGV preservation or reconstruction to mitigate adverse outcomes. Preoperative 3D-CT and MRCP facilitated surgical planning in some studies. Results showed that the LGV plays a critical role in gastric venous drainage. Its anatomical variability and inconsistent intraoperative assessment may contribute to complications. While preservation or reconstruction appears beneficial in select cases, current evidence remains limited. Further prospective, multicentre studies are essential to establish evidence-based guidelines for LGV management during pancreatectomy.

## Introduction and background

Pancreatectomy is the gold standard approach for treating pancreatic cancer. Despite its benefits, the procedure is associated with several postoperative complications. In particular, this can involve gastrointestinal disturbances and delayed gastric emptying (DGE) [1]. One important contributor to these complications is the disruption of the left gastric vein (LGV). This may occur during distal pancreatectomy or manipulation of adjacent vascular structures, including the splenic, portal and short gastric veins [2]. To achieve adequate exposure or ensure oncological clearance, LGV ligation or sacrifice may be necessary during pancreatectomy. This is especially important in cases of close tumour proximity, significant inflammation or variant venous drainage patterns [3].

The LGV plays a critical role in draining blood from the lesser curvature of the stomach into the portal vein (PV). Injury can result in gastric venous congestion (GVC), an under-recognised complication characterised by impaired venous outflow and elevated intragastric pressure [2-4]. The resulting increase in intravascular pressure therefore raises the risk of gastric bleeding, ischaemia, necrosis, and DGE. GVC has been reported in 5% to 28% of patients following pancreatectomy [4].

The degree of compensation depends on several factors, including individual venous architecture and baseline portal pressure. The presence and degree of collateral circulation from the right gastric or gastroepiploic veins also remain relevant [5].

Previous studies have linked LGV disruption with segmental portal hypertension and variceal bleeding [3,4]. As a result, these complications can often prolong recovery and postpone critical therapies, such as adjuvant chemotherapy [3-5]. Preservation and reconstruction of the LGV have been proposed to mitigate complications such as GVC. Such techniques may help maintain adequate gastric outflow. Preoperative imaging tools, including three-dimensional computed tomography (3D-CT) and intraoperative indocyanine green (ICG) fluorescence, have been proposed as a means of assisting in visualising venous anatomy and guiding intraoperative decisions [6,7].

The LGV exhibits anatomical variation in its termination, draining into the PV, splenic vein, or splenomesenteric junction. These variations can complicate surgical planning and necessitate an individualised approach. Tailoring the surgical technique to each patient's anatomy may be a means of reducing postoperative complications [2,7].

This review aims to synthesise current evidence on the role of the LGV in gastrointestinal outcomes following pancreatectomy, with particular focus on anatomical variation, imaging modalities, and intraoperative management strategies. Variations of LGV anatomy are considered, as well as intraoperative techniques aimed at preserving and reconstructing the LGV.

## Review

Methods

Two authors independently conducted a systematic search of online databases, including PubMed, MEDLINE, and Google Scholar, using relevant terms for LGV and pancreatectomy. Table 1 fully lists the inclusion and exclusion criteria. The inclusion criteria were case reports or case series that reported intraoperative or postoperative complications and perioperative identification and reconstruction of LGV in the context of pancreatectomy. Age, sex, or language restrictions were not applied. All types of pancreatectomies were included. Complications related to LGV during other forms of operation, such as gastrectomy, splenectomy, or trauma, were excluded. Combinations of the following search terms were used: "left gastric vein", "gastric vein", "total pancreatectomy", "pancreatectomy", "pancreaticoduodenectomy", "splenopancreatectomy", and "subtotal pancreatectomy". Reference lists of relevant studies were reviewed manually for potentially eligible studies.

**Table 1:** Study inclusion and exclusion criteria ICG, indocyanine green; LGV, left gastric vein

Category	Inclusion Criteria	Exclusion Criteria
Study Type	Case reports and case series involving LGV management during pancreatectomy.	Review articles, editorials, letters, animal studies, and cadaveric-only anatomical studies.
	Technical notes or surgical technique papers explicitly addressing LGV identification, preservation, ligation, or reconstruction during pancreatic surgery.	
		Conference abstracts or non-peer-reviewed literature lacking sufficient data.
	Anatomical studies based on human clinical cases with intraoperative or imaging evaluation of LGV.	
Patient Population	Human subjects of any age and sex undergoing any form of pancreatectomy (e.g., total pancreatectomy, pancreaticoduodenectomy, distal pancreatectomy, radical antegrade modular pancreatosplenectomy).	Patients undergoing surgeries unrelated to pancreatic resection (e.g., isolated gastrectomy, splenectomy, trauma cases).
		Cases involving LGV mentioned without any clinical or surgical relevance.
Surgical Context	Surgeries involving explicit identification, ligation, preservation, or reconstruction of the left gastric vein.	Studies where the LGV is not mentioned, or its course/management is not described in the context of pancreatectomy.
	Reports with documentation of LGV-related intraoperative or postoperative complications.	Procedures where LGV manipulation was not linked to any intraoperative decision-making or outcomes.
Outcomes Reported	Description of complications (e.g., gastric venous congestion, delayed gastric emptying, gastric ischaemia or bleeding) directly associated with LGV manipulation.	Studies not reporting clinical outcomes related to LGV handling.
	Intraoperative strategies to address LGV injury or congestion (e.g., venous anastomosis, gastrectomy).	Articles focused purely on LGV anatomy without clinical correlates.
Language and Accessibility	Articles in any language with available full text (translations obtained where required).	Studies where full-text access was unavailable or data extraction was not possible even after reasonable efforts.
Imaging and Intraoperative Assessment	Use of imaging (CT, MRI, 3D reconstruction) or intraoperative techniques (ICG, Doppler, ultrasound) to assess LGV anatomy or function.	Studies where imaging or intraoperative findings related to LGV were not discussed or documented.

Titles and abstracts of the selected articles were screened independently by two authors, and the full texts of potentially eligible articles were retrieved. Disagreements were resolved through consensus or consultation with the senior author.

Two authors independently extracted data using a Microsoft Excel spreadsheet (Microsoft® Corp., Redmond, WA). Information collected from each article included the author's name, year of publication, patient demographics, operation, complications related to LGV, and intervention performed.

Due to the heterogeneity of included studies (primarily case reports and case series), a formal meta-analytic risk of bias tool was not appropriate. However, to ensure consistency in assessing methodological quality, we applied the Joanna Briggs Institute (JBI) Critical Appraisal Checklist for Case Reports to evaluate individual studies [8]. Each included case was assessed independently by two reviewers, with disagreements resolved through discussion.

Results

**Figure 1:**
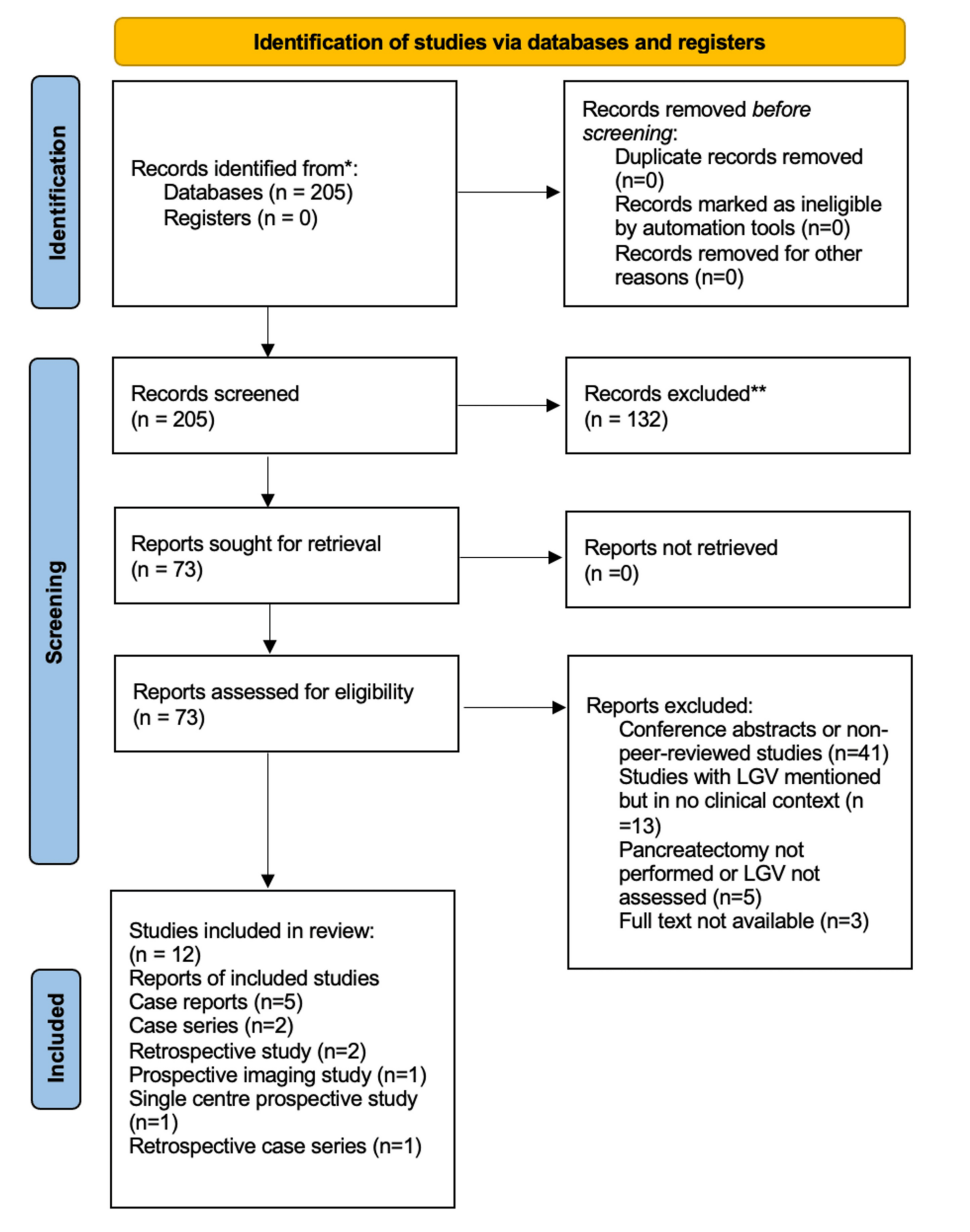
Preferred Reporting Items for Systematic Reviews and Meta-Analyses (PRISMA) 2020 flowchart showing study selection process

A total of 205 studies were identified by systematically searching the abovementioned databases. Screening titles and abstracts and removing duplicates excluded 132 studies, leaving 73 for further review. Full manuscripts were then assessed against the eligibility criteria, resulting in 12 studies [4-15] being included in this review. Studies included five case reports, two case series, two retrospective studies, one retrospective case series, one prospective imaging study, and one single-centre prospective observational study. The study selection process is detailed in the Preferred Reporting Items for Systematic Reviews and Meta-Analyses (PRISMA) flow diagram (Figure 1). Studies that failed to address the course of the LGV or did not explicitly mention the vein were excluded from this review. Details of the study characteristics can be found in Table 2. All included studies were assessed using the JBI Critical Appraisal Checklist for Case Reports [8]. Most met the majority of quality criteria, with common limitations including unclear consecutive inclusion and incomplete demographic reporting in a few cases. Overall, methodological quality was moderate to high. Quality assessment details are provided in the Appendices.

**Table 2:** Study characteristics

	Year	Number of Patients	Age	Gender	Operation	Complications	Study Design	Country
Mehrabi et al. [3]	2021	20	Not specified	Not specified	Total pancreatoduodenectomy	Not specified	Single-centre prospective nonrandomised observational study	Germany
Stoop et al. [4]	2023	268	59-74 years	125 females, 143 males	Total pancreatectomy	Not specified	Retrospective single-centre study	Sweden
Nakamura et al. [6]	2023	5	73, 60, 77, 65, 64 years	4 males, 1 female	Total pancreatectomy	One patient required subtotal gastrectomy due to lack of remaining gastric drainage vein	Case series	Japan
Rebibo et al. [7]	2012	86 (20 underwent surgery)	Not specified	Not specified	1 TP, 12 pylorus-preserving PDs, 7 left splenopancreatectomies	Injury to the left gastric vein in two patients (during left pancreatectomy and pylorus-preserving pancreaticoduodenectomy)	Prospective imaging study with surgical correlation	France
Fernández-Placencia et al. [9]	2024	1	49 years	Female	Radical antegrade modular pancreatosplenectomy (RAMPS) for resectable pancreatic ductal adenocarcinoma	Persistent congestion of the lesser curvature treated with tension-free anastomosis; intra-abdominal collection 10 days after surgery, managed with antibiotics	Case report	Peru
Yamanaka et al. [10]	2024	1	65 years	Female	Total pancreatectomy for intraductal papillary mucinous neoplasm	Not specified	Case report	Japan
Reddy et al. [11]	2023	3	52, 66, 49 years	2 males, 1 female	Total pancreatectomy, total pancreatectomy with splenectomy, pancreaticoduodenectomy	Delayed gastric emptying	Case series	India
Kimura et al. [12]	2022	105	All over 20 years	Not specified	Subtotal stomach-preserving pancreaticoduodenectomy	Delayed gastric emptying	Retrospective study	Japan
Kagota et al. [13]	2020	1	60 years	Female	Subtotal stomach-preserving pancreaticoduodenectomy	None	Case report	Japan
Üstüner et al. [14]	2020	1	65 years	Female	Pancreaticoduodenectomy	None	Case report	India
Nakao et al. [15]	2018	38	Mean 60.9 years (39-78 years)	20 males, 18 females	TPDG (16), PPTP (9), SSPTP (8), TPSD (5)	Bleeding from the nasogastric tube; gastric venous congestion	Retrospective case series	Japan
Saundroussi et al. [16]	2010	1	42 years	Female	Pancreaticoduodenectomy with en bloc resection of superior mesenteric vein, splenic vein, and portal vein confluence	Not specified	Case report	Canada

Anatomy

Eight studies reported LGV anatomy, including variations in its course and termination. Fernández-Placencia et al. and Kagota et al. utilised contrast-enhanced computed tomography (CT) imaging preoperatively to map pancreatic tumours and associated vascular anatomy [9,13], including the LGV. Kimura et al. [12] comprehensively classified the LGV’s anatomical variations using perioperative and seven-day postoperative CT imaging. The study identified four distinct anatomical types of the LGV based on its course relative to the common hepatic artery (CHA), splenic artery (SpA), and its termination site at either the PV or splenic vein (SPV). Type 1 anatomy, where the LGV coursed dorsally to the CHA or SpA and terminated at the PV, was the most observed in 47% of patients. Type 2, representing 23% of cases, described a dorsal course relative to the CHA or SpA with termination at the SPV. Type 3 involved a ventral course relative to the CHA or SpA with termination at the PV, observed in 12% of the cohort. Finally, type 4, accounting for 23% of cases, described a ventral course relative to the CHA or SpA with termination at the SPV.

Advanced imaging techniques, such as 3D-CT, were used in two studies [6,7]. Nakamura et al. [6] utilised 3D-CT imaging to assess LGV patency and identify alternative venous drainage routes in cases of LGV absence. Similarly, Rebibo et al. [7] combined standard CT imaging with 3D reconstruction to classify the LGV’s termination into three categories: termination at the PV, the superior mesenteric trunk (SMT), or the SPV. Additionally, one study [12] adopted a multimodal imaging approach, incorporating magnetic resonance cholangiopancreatography (MRCP) and triphasic contrast-enhanced CT to evaluate the LGV in the context of intraductal papillary mucinous neoplasm (IPMN). Preoperative imaging in other studies, including those by Saundroussi et al. [16] and Yamanaka et al. [10], focused on identifying the LGV’s course and relation to adjacent structures to optimise surgical planning. Furthermore, two studies [4,15] reported using abdominal imaging but did not provide specific details regarding the LGV’s anatomy.

Operative Findings

Five studies described LGV identification intraoperatively using either preoperative imaging correlation or direct visualisation. In one study [9], the LGV anatomical course was confirmed by correlating preoperative imaging with intraoperative visualisation, emphasising the value of multimodal assessment.

Other studies relied on direct visualisation for intraoperative LGV identification. Three studies [4,14,15] reported the identification and isolation of the LGV during surgery without adjunct techniques. On the other hand, Yamanaka et al. [10] employed intraoperative ultrasound to confirm its location relative to the tumour. Saundroussi et al. [16] encountered and divided the LGV during the mobilisation of the celiac vessels.

Fernández-Placencia et al. [9] visualised the LGV intraoperatively; however, their study did not report specific techniques for LGV identification. Kimura et al. performed detailed intraoperative assessments, correlating these findings with preoperative imaging to refine their classification of LGV anatomy [12].

Complications

Six studies reported complications related to LGV sacrifice. Rebibo et al. [7] reported a 10% risk of injury to the LGV during left pancreatectomy and pancreaticoduodenectomy. Moreover, three studies [7,10,12] reported intraoperative complications ranging from gastric congestion to gastric necrosis. Different techniques have been used to address this, such as re-implanting the LGV with gastric veins [10,12] and subtotal gastrectomy [7].

Regarding postoperative complications linked to LGV ligation, Reddy et al. reported a patient with mild DGE (grade A) after LGV reconstruction [11]. In Kimura et al. case series of 105 patients [12], DGE was reported in 20%, and there was a significant association between LGV ligation and DGE.

Discussion

Anatomical Variability and Imaging

An understanding of LGV anatomy is essential for surgical decision-making, given its variable course and drainage patterns.

**Figure 2:**
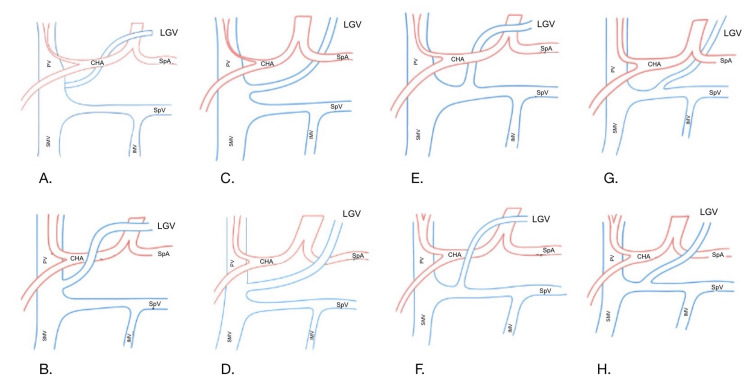
Anatomical variations of the LGV A. Left gastric vein (LGV) running posterior to common hepatic artery (CHA) joining the portal vein (PV); B. LGV running anterior to CHA joining the PV; C. LGV running posterior to splenic artery (SpA) joining the PV; D. LGV running anterior to SpA joining the PV; E. LGV running posterior to CHA joining the splenic vein (SpV); F. LGV running anterior to CHA joining the SpV; G. LGV running posterior to SpA joining the SpV; H. LGV running anterior to SpA joining the SpV. Adapted and redrawn from Kimura et al. under a Creative Commons Attribution 4.0 International License [12]. Figure credits: Vandana B. Giriradder. Hand-drawn by the author and digitised using Goodnotes software (Goodnotes, London, UK).

The classification proposed by Kimura et al. emphasises the necessity of preoperative vascular mapping [12]. Recognising predominant drainage patterns (such as type 1) can aid in anticipating vascular challenges. Figure 2 illustrates the anatomical variations of the LGV as classified by Kimura et al., depicting eight subtypes (1a-4b) based on their drainage site and relationship to the CHA [12].

Miao et al. further demonstrated a potential association between altered LGV drainage and gastric variceal bleeding in patients with segmental portal hypertension, suggesting that detailed preoperative venous assessment may have prognostic value [17]. Advancements in preoperative imaging, particularly 3D-CT and MRCP, have improved the visualisation of venous drainage patterns [18]. These tools allow for individualised surgical planning and risk stratification.

Intraoperative Strategies

An evolving surgical philosophy that places more emphasis on functional outcomes than en bloc resection alone is reflected in the shift toward venous preservation and reconstruction techniques, such as gastroepiploic vein anastomoses or LGV-to-adrenal vein anastomoses [8,19].

The lack of standardised protocols is indicated by the variation in approach, even though the majority of studies used direct visualisation or correlation with preoperative imaging to detect the LGV intraoperatively. Real-time flowmetry and ICG fluorescence imaging are two examples of emerging technologies that could provide more objective evaluations of gastric venous drainage during surgery. The ability of these intraoperative adjuncts to direct intraoperative choices and maintain stomach viability is demonstrated by the GENDER trial [3].

Postoperative Complications

While these studies do not isolate LGV injury as a variable, our review suggests it may be an under-recognised contributor, particularly when alternative venous outflow is inadequate. Furthermore, LGV ligation may clinically increase the risk of gastric varices and segmental portal hypertension [20].

The relationship between LGV injury and DGE, as seen in multiple studies, suggests that the LGV plays a pivotal role in maintaining adequate venous return from the stomach. DGE was associated with LGV ligation and occurred in 20% of patients in the series by Kimura et al. [12]. This is in line with meta-analysis by Pang et al., who found a number of risk factors for DGE following distal pancreatectomy, including disruption of stomach drainage and venous congestion [21]. DGE rates following pancreatoduodenectomy and distal pancreatectomy have also been reported in larger evaluations to be 12.7% and 9.5%, respectively [22,23].

Study Limitations

While the cumulative data support the preservation or reconstruction of the LGV, most available studies are limited to case reports or small series. This heterogeneity limits the generalisability of findings. Moreover, comparing LGV-related outcomes across different surgical procedures (e.g., PD vs. total pancreatectomy) is inherently challenging due to differences in anatomical dissection fields, surgical goals, and patient profiles. Several studies lacked detailed intraoperative or imaging-based anatomical descriptions of the LGV, which may have led to underrepresentation of clinically significant variations or complications.

There is also a paucity of prospective or randomised studies directly comparing various reconstruction techniques or evaluating their long-term outcomes. Standardised outcome reporting and stratification by surgical type are necessary for meaningful analysis.

These limitations underscore the need for prospective, multi-institutional studies with standardised protocols and validated outcome measures to better define the role of LGV preservation and reconstruction in pancreatic surgery.

The quality of all included studies was assessed using the JBI Critical Appraisal Checklist for Case Reports [8]. Most studies fulfilled the majority of the criteria, with overall methodological quality rated as moderate to high. Common limitations included unclear reporting of consecutive inclusion and, in one case, incomplete demographic information. Full quality assessment results are presented in the Appendices.

Future Directions

Future research should focus on the standardisation of LGV management protocols during pancreatic resections. High-resolution, AI-assisted vascular mapping could be integrated into preoperative imaging workflows to enhance risk prediction for venous congestion [24]. Intraoperatively, real-time ICG fluorescence and Doppler ultrasonography should be employed systematically to evaluate LGV patency and collateral flow [25].

Additionally, comparative trials of venous reconstruction strategies, such as LGV-to-adrenal or LGV-to-RGEV anastomoses, are needed to establish optimal techniques. These studies should include validated endpoints such as rates of DGE, gastric ischemia, and quality-of-life outcomes. The development of procedural checklists or intraoperative decision trees based on LGV anatomy could further refine practice and reduce variability in outcomes.

## Conclusions

This systematic review establishes a foundation for examining the role of the LGV in pancreatectomy. The findings demonstrate that disruption of the LGV can lead to complications that can delay patient recovery. Preservation or reconstruction of the LGV appears to improve outcomes in selected cases, especially where alternative venous drainage was limited. The evidence, however, is limited by small, retrospective studies with a lack of control groups. Significant heterogeneity in surgical techniques and outcome reporting further limits the generalisability of our findings. Nevertheless, this review does underscore the importance of detailed anatomical assessment of the LGV in reducing morbidity. Future research should focus on multicentre, randomised controlled trials with long-term outcomes to inform evidence-based surgical guidelines for LGV in the context of pancreatectomy.

## Appendices

**Table 3:** Joanna Briggs Institute (JBI) Critical Appraisal of included case reports “Yes” indicates the criterion was met. “No” indicates it was not met. “Unclear” denotes insufficient information. Quality was assessed using the Joanna Briggs Institute (JBI) Critical Appraisal Checklist for Case Reports.

Author	Year	Inclusion criteria clearly stated?	Condition measured reliably?	Valid identification methods used?	Consecutive inclusion of participants?	Complete inclusion of participants?	Demographics clearly reported?	Clinical info clearly reported?	Outcomes or follow-up reported?	Site/clinic demographics reported?	Statistical analysis appropriate?	Notes
Mehrabi et al. [3]	2021	Yes	Yes	Yes	Unclear	Yes	Yes	Yes	Yes	Yes	Yes	The study does not explicitly state if inclusion was consecutive; likely due to pilot nature.
Stoop et al. [4]	2023	Yes	Yes	Yes	Yes	Yes	Yes	Yes	Yes	Yes	Yes	-
Nakamura et al. [6]	2023	Yes	Yes	Yes	Unclear	Yes	Yes	Yes	Yes	Yes	Yes	Not stated whether participants were enrolled consecutively
Rebibo et al. [7]	2012	Yes	Yes	Yes	Yes	Yes	Unclear	Yes	Yes	Yes	Yes	Demographic details not mentioned. Clinical focus on imaging data.
Fernández-Placencia et al. [9]	2024	Yes	Yes	Yes	Yes	Yes	Yes	Yes	Yes	Yes	Yes	-
Yamanaka et al. [10]	2024	Yes	Yes	Yes	Yes	Yes	Yes	Yes	Yes	Yes	Yes	-
Reddy et al. [11]	2023	Yes	Yes	Yes	No	No	Yes	Yes	Yes	Yes	Not applicable	The paper presents selected illustrative cases, not a full consecutive series.
Kimura et al. [12]	2022	Yes	Yes	Yes	Yes	Yes	Yes	Yes	Yes	Yes	Yes	-
Kagota et al. [13]	2020	Yes	Yes	Yes	Yes	Yes	Yes	Yes	Yes	Yes	Yes	-
Üstüner et al. [14]	2020	Yes	Yes	Yes	Yes	Yes	Yes	Yes	Yes	Yes	Yes	-
Nakao et al. [15]	2018	Yes	Yes	Yes	Yes	Yes	Yes	Yes	Yes	Yes	Yes	-
Saundroussi et al. [16]	2010	No	Yes	Yes	No	Yes	Yes	Yes	Yes	Yes	Not applicable	Single-case, consecutive inclusion not applicable.
